# Association of polygenic risk scores with Alzheimer's disease and plasma biomarkers among Chinese older adults: A community‐based study

**DOI:** 10.1002/alz.13924

**Published:** 2024-08-22

**Authors:** Tingting Hou, Keke Liu, Wenxin Fa, Cuicui Liu, Min Zhu, Xiaoyan Liang, Yifei Ren, Shan Xu, Xiang Wang, Shi Tang, Yongxiang Wang, Lin Cong, Qihuan Tan, Yifeng Du, Chengxuan Qiu

**Affiliations:** ^1^ Department of Neurology Shandong Provincial Hospital Affiliated to Shandong First Medical University Jinan Shandong P.R. China; ^2^ Department of Neurology Shandong Provincial Hospital Shandong University Jinan Shandong P.R. China; ^3^ Shandong Provincial Clinical Research Centre for Neurological Diseases Jinan Shandong P.R. China; ^4^ Department of Neurology, Key Laboratory of Endocrine Glucose & Lipids Metabolism and Brain Aging Ministry of Education Shandong Provincial Hospital Affiliated to Shandong First Medical University Jinan Shandong P.R. China; ^5^ Institute of Brain Science and Brain‐Inspired Research Shandong First Medical University & Shandong Academy of Medical Sciences Jinan China; ^6^ Department of Neurobiology Care Sciences and Society, Aging Research Center and Center for Alzheimer Research Karolinska Institute‐Stockholm University Solna Sweden; ^7^ Department of Public Health Epidemiology and Biostatistics University of Southern Denmark Odense Denmark

**Keywords:** Alzheimer's disease, plasma Alzheimer's biomarkers, polygenic risk score, population‐based study

## Abstract

**INTRODUCTION:**

We examined the associations of polygenic risk score (PRS) with Alzheimer's disease (AD) and plasma biomarkers in the Chinese population.

**METHODS:**

This population‐based study used baseline data from MIND‐China (2018; *n* = 4873) and follow‐up data from dementia‐free individuals (2014–2018; *n* = 2117). We measured AD‐related plasma biomarkers in a subsample (*n* = 1256). Data were analyzed using logistic and Cox regression models.

**RESULTS:**

We developed PRS with (PRS*_APOE_*) and without (PRS_non‐_ *_APOE_*) apolipoprotein E *(APOE)* gene. In the longitudinal analysis, PRS*_APOE_* was associated with a multivariable‐adjusted hazards ratio of 1.91 (95% CI = 1.13–3.23) for AD. PRS*_APOE_* in combination with demographics yielded discriminative (area under the curve [AUC]) and predictive(C‐statistic) accuracy of 0.80 (95% confidence interval [CI] = 0.77–0.84) and 0.80 (0.77–0.82), respectively. PRS_non‐_ *_APOE_* showed an association with AD risk similar to PRS*_APOE_*. PRS*_APOE_*, but not PRS_non‐_ *_APOE_*, was associated with reduced plasma Aβ42/Aβ40 ratio and increased Neurofilament light chain (NfL) (*p* < 0.05).

**DISCUSSION:**

The PRS with and without *APOE* gene, in combination with demographics, shows good discriminative and predictive ability for AD. The AD‐related pathologies underlie AD risk associated with PRS*_APOE_*.

**Highlights:**

- The PRS*_APOE_* and PRS_non‐_ *_APOE_* were associated with AD risk in the Chinese population.
- The PRS*_APOE_* and PRS_non‐_ *_APOE_*, in combination with demographics, showed good discriminative and predictive ability for AD.
- The AD‐related pathologies underlie the AD risk associated with PRS*_APOE_* but not PRS_non‐_ *_APOE_*.

## 1 BACKGROUND

Alzheimer's disease (AD) accounts for up to 75% of all cases of clinically diagnosed dementia, with the vast majority of AD cases being late‐onset AD.^1^ The risk of late‐onset AD is determined by the complex interplay of lifelong genetic and environmental factors, with the heritability being up to 79%.^2^ Apolipoprotein E *(APOE*) ε4 allele is the strongest genetic risk factor for late‐onset AD, which could explain ∼20% of the AD risk.^3^ Apart from *APOE* ε4 allele, a series of genome‐wide association studies (GWASs) have identified a large number of single nucleotide polymorphisms (SNPs) that are related to AD.^4^, ^5^ However, these individual SNPs generally confer minor risk for AD that is often negligible. The polygenic risk score (PRS) allows to combine a large number of risk alleles potentially associated with AD for estimating genetic risk for AD.^6^

Previous studies have developed various PRSs for AD and used the PRSs to predict lifetime AD risk, although the prediction accuracy varied substantially across studies of ethnically diverse populations (C‐statistic range: 0.57–0.84).^7^, ^8^, ^9^, ^10^ Moreover, studies have suggested that these PRSs are correlated with AD biomarkers in central nervous system such as Aβ42 in cerebrospinal fluid (CSF), Aβ deposition in the brain, and hippocampal atrophy^7^, ^11^, ^12^, indicating that PRS can modulate the Alzheimer pathology. However, most of the previous studies that investigate PRS for AD have been conducted among Caucasians within the International Genomics of Alzheimer's Project (IGAP).^5^ Current studies concerning PRS for AD in Chinese people have been conducted in the clinical settings^13^, ^14^ but not in the general population.

In this community‐based study, we sought to explore the cross‐sectional and longitudinal associations of PRS with AD in Chinese older adults while taking into account *APOE* gene, and further to assess the discriminative and predictive ability of the PRS for AD. In addition, we further examined the associations of the PRS with AD‐related plasma biomarkers to facilitate the interpretation of the PRS‐AD associations.

## 2 METHODS

### 2.1 Study design and participants

**FIGURE 1:**
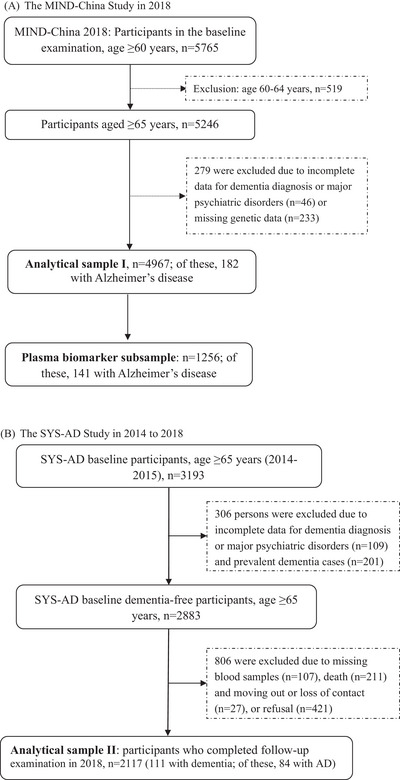
Flowcharts of study participants in (A) the MIND‐China Study in 2018 and (B) the SYS‐AD Study in 2014 to 2018. AD, Alzheimer's disease; MIND‐China, Multimodal Interventions to Delay Dementia and Disability in Rural China; SYS‐AD, the Shandong Yanggu Study of Aging and Dementia

This population‐based study used data from two waves of a survey conducted among rural older adults who were living in the same geographical region, covering 52 villages (rural communities) of Yanlou Town, Yanggu County, western Shandong Province, that is, the Multimodal Interventions to Delay Dementia and Disability in rural China (MIND‐China) in 2018 and the Shandong Yanggu Study of Aging and Dementia (SYS‐AD) in 2014–2015.^15^ Figure 1 shows the flowchart of the study participants in the two waves of examination. (1) MIND‐China: In March–September 2018, we conducted the survey as part of baseline assessments of the MIND‐China study that targeted people who were aged 60 years and older by the end of 2017^16^. A total of 5765 residents (74.9% of all eligible people) were examined for MIND‐China. Of these, we excluded 519 persons who were aged 60–64 years. Of the remaining 5246 participants who were aged ≥65 years, 279 were excluded due to major psychiatric disorders or missing dementia diagnosis (*n* = 46) and missing genetic data (*n* = 233), leaving 4967 (86.2%) participants for analyzing the cross‐sectional association between PRS and AD (analytical sample I). Of these, data on plasma neuropathological biomarkers were available in 1256 individuals. This subsample was used for the analysis of the cross‐sectional association between PRS and AD‐related plasma biomarkers (Figure 1A). (2) SYS‐AD: SYS‐AD was initiated in 2014‐2015 (baseline) that engaged people who were aged 65 years and older in Yanlou Town.^17^ A total of 3193 participants were examined via face‐to‐face interviews, clinical examination, and testing at baseline of SYS‐AD. Of these, 310 were excluded due to prevalent dementia that was diagnosed according to the DSM‐IV criteria (*n* = 201) or missing dementia diagnosis or major psychiatric disorders (*n* = 109), leaving 2883 persons who were free of dementia at baseline (2014–2015). Of these, 806 persons were excluded due to missing genetic data (*n* = 107), death prior to the follow‐up examination in 2018 (*n* = 211), moving out or loss of contact (*n* = 27), or refusal to the follow‐up examination (*n* = 421), leaving 2117 participants who were free of dementia at baseline in 2014 and who had complete follow‐up data in 2018 for analyzing the longitudinal association between PRS and AD (analytical sample II). Of these 2117 participants, 2078 were followed up as part of the MIND‐China baseline assessments in 2018, and an additional 39 were followed up separately (Figure 1B).

**RESEARCH IN CONTEXT**

1. **Systematic review**: We searched PubMed for literature investigating the association of polygenic risk score (PRS) with Alzheimer's disease (AD) and AD‐related biomarkers. The majority of population‐based studies that examine the association of PRS with AD have been conducted among Caucasian and Black populations, and a few studies have examined the association of PRS with AD biomarkers. A few studies from China have explored the association of PRS with AD in the clinical settings. Studies that develop the PRS with and without apolipoprotein E gene for AD and examine its association with AD‐related plasma biomarkers in the general Chinese population are currently lacking.
2. **Interpretations**: Our population‐based cohort study of Chinese older adults revealed that a higher PRS was associated with a greater risk of AD. PRS*_APOE_* and PRS_non_ *_‐APOE_*, in combination with demographics, shows good discriminative and predictive ability for AD. The AD‐related pathologies may underlie the risk of AD associated with PRS*_APOE_*.
3. **Future directions**: Future large‐scale population‐based cohort studies that integrate clinical and comprehensive genetic data with sensitive plasma AD biomarker data (e.g., phosphorated‐tau proteins) are warranted to replicate the PRS for AD in the general Chinese populations and to facilitate the interpretations.

### 2.2 Data collection at baseline and follow‐up examinations

In both the MIND‐China and SYS‐AD studies, data were collected by trained staff following structured questionnaires through face‐to‐face interviews, clinical examinations, cognitive testing, and laboratory tests.^15^, ^16^ We collected data on demographic factors (e.g., age, sex, and education), lifestyle factors (e.g., alcohol consumption, smoking, and leisure‐time physical activity), health conditions (e.g., hypertension, diabetes, and coronary heart disease), use of medications (e.g., antihypertensive and hypoglycemic agents), and cognitive function (e.g., the Mini‐Mental State Examination [MMSE]). Alcohol consumption and smoking status were categorized as current, former, and never drinking or smoking. Hypertension was defined as arterial blood pressure ≥140/90 mmHg or current use of antihypertensive agents (ATC codes C02, C03, and C07‐C09). Diabetes was defined as fasting blood glucose ≥7.0 mmol/L, taking antidiabetic agents (ATC code A10) or having a self‐reported diabetes history. Dyslipidemia was defined as TC ≥ 6.2 mmol/L, or TG ≥ 2.3 mmol/L, or LDL‐C ≥ 4.1 mmol/L, or HDL‐C < 1.0 mmol/L, or use of hypolipidemic agents (ATC code C10). Coronary heart disease was defined according to self‐reported history or ECG examination, including angina, myocardial infarction, coronary angioplasty, and coronary artery bypass grafting. The history of clinical stroke was ascertained according to self‐reported history and neurological examination.

### 2.3 Diagnosis of dementia and AD

In both the MIND‐China study (2018) and the SD‐YSAD study (2014–2015), dementia was diagnosed following the same criteria outlined in the Diagnostic and Statistical Manual of Mental Disorders, Fourth Edition (DSM‐IV)^18^, in which a three‐step diagnostic procedure was followed, as previously described.^19^ ^.^ In brief, the trained interviewers and examining physicians made a preliminary judgment for participants who were suspicious of having cognitive impairment or dementia according to their performances during the interviews and neuropsychological assessments. Then, senior neurologists, who specialized in dementia care and treatment, made a preliminary diagnosis of dementia by reviewing all records with regard to functional, medical, neurological, psychiatric, and neuropsychological data. Finally, the neurologists conducted the second face‐to‐face interviews with participants who received a preliminary diagnosis of dementia or who had insufficient information for the diagnosis of dementia and informants (e.g., family members, neighbors, or village doctors who provided routine primary care for local residents), and reassessed their medical history, cognitive status, activities of daily living (ADLs), and whenever available, neuroimaging data. Following all the interviews and assessments, a panel of neurologists specializing in dementia diagnosis and care at Shandong Provincial Hospital made the final diagnosis of dementia. AD was diagnosed according to the National Institute on Aging‐Alzheimer's Association (NIA‐AA) criteria for probable AD dementia.^20^

### 2.4 Genotyping and selection of SNPs

According to the GWASs, WGS, and large‐scale studies,^4^, ^5^, ^21^, ^22^, ^23^ we selected 53 single nucleotide polymorphisms (SNPs) from 33 candidate genes for the development of PRS for AD (Table S1). The genotyping procedure was as follows.^24^ genomic DNA was extracted from venous blood leukocytes using the TIANamp blood DNA kit (Tiangen, Beijing, China) following the manufacturer's instructions. Then, DNA was quantified using Qubit 3.0, and a total amount of 100 ng genomic DNA per sample was used as input material for the DNA library preparations. Multiplex PCR primers were designed to capture all SNPs included in our study (http://www.igenetech.com/design). Sequencing libraries were generated using MultipSeqCustom Panel (iGeneTech, Beijing, China) following the manufacturer's recommendations and index codes were added to each sample. Finally, qualified libraries were subjected to the next‐generation sequencing (NGS) on a NovaSeq system (Illumina), and raw data were filtered to remove low‐quality reads using the FastQC. Genotyping was conducted by an operator who was blinded to the clinical data. We then verified the results of the *APOE* genotype (rs7412 and rs429358) in 5% of the sample by using the Sanger sequencing methods. The two methods yielded identical results.

### 2.5 PRS

The SNP alleles were excluded if the minor allele frequency (MAF) of the SNPs was < 0.01 or did not meet Hardy‐Weinberg equilibrium at *p* < 0.05. Detailed selection and exclusion processes are provided in Table S1. The 2018 MIND‐China data were used to develop the PRS for AD. We compared the frequencies of SNP allele between individuals with and without AD in the 2018 MIND‐China participants. We used logistic regression models to estimate odds ratios (ORs) of AD associated with risk alleles (vs. common genotype) controlling for age and sex.^25^ The SNP alleles that were associated with elevated ORs of AD at *p* < 0.30 were selected for the PRS construction. Out of the 53 SNPs, 14 genetic variants and *APOE* gene were included in the weighted PRS (PRS*_APOE_*). The PRS was calculated as the sum of the products of SNP dosages of the included genetic variants and their respective weights.^25^ In addition, a PRS without the *APOE* genotype (PRS_non‐_ *_APOE_*) was created to evaluate the impact of *APOE* genotype on the association of PRS with AD. We categorized all the participants according to quartiles of the PRS. The distribution of the PRS and cut‐off values of the quartiles are shown in Figure S1.

### 2.6 Plasma Aβ42, Aβ40, t‐tau, and NfL concentrations

Fasting peripheral blood samples were collected. Ethylenediaminetetraacetic acid (EDTA) plasma was sampled, aliquoted, and frozen at ‐80°C according to standard procedures. Prior to the analysis, the samples were thawed at 4°C and centrifuged at 10,000 × *g* for 10 min at room temperature. Supernatants were collected and subsequently kept on ice until analysis. Plasma Aβ42, Aβ40, and t‐tau concentrations were measured using a Human Neurology 3‐Plex A assay (N3PA) Kit. Neurofilament light chain (NfL) was measured by using the Simoa NF‐light Advantage Kit. All measurements were performed on the fully automated single molecule array (SIMOA) HD‐X analyzer (Quanterix Corp, MA, USA) according to the manufacturer's protocol (Wayen Biotechnologies Inc., Shanghai, China). All samples were measured in duplicate. Two quality control samples with expected values and a pool sample prepared by combining 20 individual healthy donor samples were run in duplicate on each plate for each analyte to control the intra‐assay coefficients of variation (CV). The upper and lower detection limits for quality sample of each index were within the range indicated in the manual. The intra‐assay CVs for the pool sample were well below the accepted cutoff of 20% (12.7% for plasma Aβ40, 6.8% for plasma Aβ42, 11.2% for plasma t‐tau, and 6.7% for plasma NfL). The average inter‐assay CV of the pool sample was 2.9% for plasma NfL, 2.6% for Aβ40, 3.5% for Aβ42, and 3.3% for t‐tau.

### 2.7 Statistical analysis

We presented the mean (standard deviation) for continuous variables and frequency (%) for categorical variables. *t*‐Test or the Mann‐Whitney *U*‐test for continuous variables and chi‐squared tests for categorical variables were used to test the differences in characteristics between participants without and with AD. We presented minor allele frequencies (MAF) for all SNPs in participants of the 2018 MIND‐China examination by AD status. In the 2018 MIND‐China participants, we used the logistic regression model to examine the cross‐sectional association between PRS and AD, in which PRS was analyzed as both a continuous and a categorical (quartiles) variable. Then, we used the general linear regression models to examine the cross‐sectional associations between the PRS and AD‐related plasma biomarkers in the subsample. In the SD‐SYAD dementia‐free cohort at baseline (2014), we used Cox proportional hazard models to examine the association between PRS and incident AD diagnosed in 2018. The discriminative ability of the PRS for AD was assessed using the area under the receiver operating characteristic curve (AUC). The C‐statistic was used to assess the predictive ability of PRS for incident AD using the SD‐SYAD follow‐up data (2014 to 2018). The agreement between the observed and predicted outcomes was evaluated with calibration plots. All measures were corrected for model optimism by internal validation with enhanced bootstrapping (100 repetitions).

The PRS*_APOE_* and PRS_non‐_ *_APOE_* were analyzed separately in association with AD and AD‐related plasma biomarkers and the prediction of incident AD. Two‐tailed *p* < 0.05 was considered statistically significant. All analyses were performed using R 3.5.0 (R Core Team, R Foundation for Statistical Computing, Vienna, Austria).

## 3 RESULTS

### 3.1 Characteristics of the study participants

In the 2018 MIND‐China survey, the mean age of the 4873 participants was 71.60 years (SD 5.36), 57.2% were female, 39.9% were illiterate (no formal school education), and 182 (3.73%) were diagnosed with AD (Table 1). Compared with participants without dementia, those with AD were older, more likely to be female and less educated, less likely to smoke and drink alcohol, and had a lower MMSE score (*p* < 0.05), but the two groups had no significant differences in the distribution of coronary heart disease and *APOE* ε4 allele (Table 1). The mean PRS*_APOE_* of all participants was 0.28 (range, ‐1.481–2.181; SD, 0.47) and the mean PRS_non‐_ *_APOE_* was 0.23 (range, ‐1.481–1.789; SD, 0.43). The scores of PRS*_APOE_* or PRS_non‐_ *_APOE_* were higher in participants with AD than those without AD (*p* < 0.01) (Table 1).

**TABLE 1:** Characteristics of study participants.

	MIND‐China 2018 (analytical sample I)				SYS‐AD 2014 (analytical sample II)			
	Total sample (*n* = 4873)^a^	Prevalent AD			Total sample (*n* = 2090)^a^	Incident AD defined in 2018		
Characteristics		No (*n* = 4691)	Yes (*n* = 182)	*p*‐Value		No (*n* = 2006)	Yes (*n* = 84)	*p*‐Value
Age, years	71.60(5.36)	71.37(5.14)	77.51(7.16)	<0.001	70.23(4.62)	70.05(4.50)	74.42(5.51)	<0.001
Female sex, *n* (%)	2789(57.23)	2651(56.51)	138(75.82)	<0.001	1248(59.71)	1177(58.67)	71(84.52)	<0.001
Illiteracy, *n* (%)	1944(39.89)	1810(38.58)	134(73.63)	<0.001	795(38.04)	732(36.49)	63(75.00)	<0.001
Current smoking, *n* (%)	1000(20.53)	984(20.98)	16(8.79)	<0.001	701(33.54)	687(34.63)	14(16.67)	<0.001
Alcohol drinking, *n* (%)	1292(26.75)	1272(27.36)	20(11.05)	<0.001	704(33.68)	686(34.56)	18(21.43)	0.013
Stroke, *n* (%)	726(14.90)	698(14.89)	28(15.38)	0.854	176(8.42)	170(8.47)	6(7.14)	0.667
Coronary heart disease, *n* (%)	1066(21.88)	1013(21.59)	53(29.12)	0.016	385(18.42)	366(18.25)	19(22.62)	0.311
Hypertension, *n* (%)	3248(66.65)	3131(67.29)	117(64.64)	0.456	1539(73.64)	1477(73.67)	62(73.81)	0.977
Diabetes, *n* (%)	678(13.91)	649(13.84)	29(15.93)	0.422	285(13.64)	279(13.91)	6(7.14)	0.077
Hypercholesterolemia, *n* (%)	1132(23.23)	1084(23.11)	48(26.37)	0.306	576(27.56)	551(27.52)	25(29.76)	0.653
MMSE score	20.68(6.14)	21.01(5.87)	9.82(4.26)	<0.001	22.01(5.34)	22.23(5.28)	16.87(4.15)	<0.001
*APOE* ε4 allele, *n* (%)	779(15.99)	745(15.88)	34(18.68)	0.312	322(15.41)	304(15.15)	18(21.43)	0.119
PRS*_APOE_*	0.28(0.47)	0.28(0.47)	0.44(0.51)	<0.001	0.28(0.47)	0.27(0.47)	0.41(0.51)	0.015
PRS_non_ *_‐APOE_*	0.23(0.43)	0.23(0.42)	0.38(0.25)	<0.001	0.23(0.43)	0.23(0.43)	0.35(0.46)	0.022

*Note*: Data are mean (SD) unless otherwise specified. *p*‐value was for test of difference between participants without and with Alzheimer's disease. In subsequent analyses, categorical variables with missing values were replaced with a dummy variable, and continuous variables with missing values were replaced with a mean value.

Abbreviations: AD, Alzheimer's disease; *APOE*, apolipoprotein E gene; MIND‐China: Multimodal Interventions to Delay Dementia and Disability in Rural China; MMSE, Mini‐Mental State Examination; PRS, polygenic risk score.; SYS‐AD: Shandong Yanggu Study of Aging and Dementia.

^a^ The number of people with missing values was 123 in MMSE score, 1 in smoke, 43 in alcohol consumption, 3 in stroke, 39 in hypertension of 2018 assessments and 22 in smoke, 21 in alcohol consumption, 1 in hypertension, 4 in hypercholesterolemia of 2014–2015 baseline assessments.

In the SYS‐SD cohort, the mean baseline age of the 2117 dementia‐free participants was 71.23 years (SD, 4.62), 59.2% were women, and 38.0% had no school education. During an average of 3.78 years (SD, 0.27) of follow‐up, 111 participants developed dementia, including 84 with AD. People who developed incident AD during the follow‐up were older, more likely to be women, drink alcohol, and smoke than those who did not (*p* < 0.05), but the two groups had no significant difference in the proportion of *APOE* ε4 allele carriers (*p* = 0.119) (Table 1).

### 3.2 Cross‐sectional association of PRS with AD in the 2018 MIND‐China Study

The PRS*_APOE_* was significantly associated with an increased likelihood of AD, even when controlling for multiple potential confounders (*p* < 0.01) (Table 2). When PRS*_APOE_* was analyzed as quartiles, the likelihood of AD was increased with increasing quartiles of PRS*_APOE_* (*p* for linear trend < 0.01). The multivariable‐adjusted OR of AD associated with participants in the fourth quartiles (Q4 vs. Q1) was 2.17 (95% CI: 1.39, 3.43). We detected a statistical interaction of PRS*_APOE_* with sex on AD risk (*p* for interaction = 0.01). Women in the fourth quartile of PRS*_APOE_* had a 7.57‐fold increased likelihood of AD (95% CI: 3.44, 19.88) compared with men in the first quartile of PRS*_APOE_*. The association of PRS_non‐_ *_APOE_* with the likelihood of AD was similar to that of PRS*_APOE_* (Table 2), but there were no statistical interactions of PRS_non‐_ *_APOE_* with sex, age, and *APOE* genotype on AD risk (*p* for all interactions > 0.05).

**TABLE 2:** Cross‐sectional association of polygenic risk score (PRS) with Alzheimer's disease: The MIND‐China Study in 2018.

PRS with and without *APOE* gene	No. of participants	No. of cases	Odds ratio (95% confidence interval)	
			Model 1^a^	Model 2^a^
PRS*_APOE_*				
Continuous	4873	182	2.15 (1.56, 2.95)^*^	2.06 (1.49, 2.84)^*^
Categorical (quartiles)				
Q1 (<‐0.030)	1228	32	1.00 (reference)	1.00 (reference)
Q2 (‐0.030–0.256)	1209	37	1.13 (0.69, 1.87)	1.14 (0.69, 1.87)
Q3 (0.256–0.556)	1218	47	1.47 (0.92, 2.37)	1.44 (0.90, 2.33)
Q4 (> 0.556)	1218	66	2.22 (1.44, 3.50)^*^	2.22 (1.39, 3.43)^*^
*p* for linear trend			<0.001	<0.001
PRS_non_ *_‐APOE_*				
Continuous	4873	182	2.35 (1.64, 3.36)^*^	2.24 (1.56, 3.22)^*^
Categorical (quartiles)				
Q1 (<‐ 0.036)	1222	30	1.00 (reference)	1.00 (reference)
Q2 (‐0.036–0.242)	1218	40	1.29 (0.79, 2.13)	1.31 (0.80, 2.18)
Q3 (0.242–0.511)	1216	44	1.40 (0.87, 2.30)	1.39 (0.86, 2.29)
Q4 (> 0.511)	1217	68	2.38 (1.53, 3.78)^*^	2.36 (1.51, 3.78)^*^
*p* for linear trend			<0.001	<0.001

Abbreviations: *APOE*, apolipoprotein E gene.; MIND‐China, Multimodal Interventions to Delay Dementia and Disability in Rural China; PRS, polygenic risk score.

^a^ Model 1 was adjusted for age and sex; model 2 was adjusted for age, sex, education, smoking, alcohol drinking, hypertension, hyperlipidemia, diabetes, coronary heart disease, and stroke.

^*^ *p *< 0.05.

### 3.3 Longitudinal association of PRS with incident AD in the SYS‐AD cohort (2014 to 2018)

The PRS*_APOE_* was significantly associated with an increased risk of incident AD, even in the fully‐adjusted model (*p* < 0.01) (Table 3). When the PRS*_APOE_* was analyzed as quartiles, participants in the fourth quartile (Q4 vs. Q1) had a nearly two‐fold increased risk of AD (hazard ratio [HR] = 1.97, 95% CI: 1.08–3.60) after adjusting for age and sex, and the association was slightly diluted and became statistically marginal after adjusting for additional potential confounders (HR = 1.92; 95% CI: 0.98–3.73; *p* = 0.068) (Table 3). The hazard ratio of AD associated with PRS_non‐_ *_APOE_* was similar to that with PRS*_APOE_*. Participants in the fourth quartile (Q4 vs. Q1) of PRS_non‐_ *_APOE_* exhibited a significantly increased risk of developing AD, even in the multivariable‐adjusted model (HR = 2.15, 95% CI: 1.10–4.21) (Table 3).

**TABLE 3:** Longitudinal association of polygenic risk (PRS) score with incident Alzheimer's disease: The SYS‐AD Study 2014–2018.

PRS with and without *APOE* gene	No. of participants	No. of cases	Hazard ratio (95% confidence interval)	
			Model 1^a^	Model 2^a^
PRS*_APOE_*				
Continuous	2090	84	1.93 (1.23, 3.03)^*^	1.91 (1.13, 3.23)^*^
Categorical (quartiles)				
Q1 (< 0.028)	525	16	1.00 (reference)	1.00 (reference)
Q2 (‐0.028–0.259)	520	19	1.08 (0.55, 2.11)	1.09 (0.56, 2.13)
Q3 (0.259–0.553)	524	18	1.10 (0.56, 2.16)	1.11 (0.56, 2.18)
Q4 (> 0.553)	521	31	1.97 (1.08, 3.60)^*^	1.92 (0.98, 3.73)
*p* for linear trend			0.022	0.068
PRS_non_ *_‐APOE_*				
Continuous	2090	84	2.00 (1.20,3.34)	1.92 (1.13, 3.26)
Categorical (quartiles)				
Q1 (<‐0.035)	526	13	1.00 (reference)	1.00 (reference)
Q2 (‐0.035–0.242)	519	22	1.55 (0.78, 3.08)	1.52 (0.76, 3.02)
Q3 (0.242–0.507)	523	21	1.45 (0.73, 2.91)	1.38 (0.69, 2.79)
Q4 (> 0.507)	522	28	2.26 (1.17, 4.37)^*^	2.15 (1.10, 4.21)^*^
*p* for linear trend			0.021	0.037

Abbreviations: *APOE*, apolipoprotein E gene.; PRS, polygenic risk score; SYS‐AD, Shandong Yanggu Study of Aging and Dementia.

^a^ Model 1 was adjusted for age and sex; model 2 was adjusted for age, sex, education, smoking, alcohol drinking, hypertension, hyperlipidemia, diabetes, coronary heart disease, and stroke.

^*^ *p *< 0.05.

### 3.4 Discriminative and predictive performance of PRS for AD

The discriminative and predictive performance of PRS alone and in combination with demographic features for AD were assessed in four models: PRS alone in model 1; PRS and age in model 2; PRS, age, and sex in model 3; and PRS, age, sex, and education in model 4 (Table 4). We assessed the ability of PRS to discriminate AD from non‐dementia by using AUC among the 2018 MIND‐China participants. The AUC of the PRS*_APOE_* alone was 0.60 (95% CI: 0.55–0.64). When age, sex, and education were added to the model step by step, the ability to discriminate AD from non‐dementia was steadily improved, with the AUC being increased from 0.76 (95% CI: 0.72–0.80) and 0.79 (0.75–0.82) to 0.80 (0.77–0.84). Using data from the SYS‐AD cohort who were followed up from 2014 to 2018, we assessed the predictive ability of PRS alone and in combination with demographics for incident AD by estimating C‐statistics. The predictive ability (C‐index) of PRS*_APOE_* alone for incident AD was 0.58 (95% CI: 0.55–0.61), and the predictive ability was significantly improved, along with addition of age, sex, and education, with the C‐index being 0.74 (95% CI: 0.72–0.77), 0.78 (0.76–0.81), and 0.80 (0.77–0.82), respectively (Table 4). The discriminative and predictive ability of PRS_non_ *_‐APOE_* for AD was similar to that of PRS*_APOE_* (Table 4).

**TABLE 4:** The performance of different models for discriminating and predicting Alzheimer's disease (AD) in cross‐sectional and longitudinal analysis.

Models	Factors	AUC (95% CI)^a^	C‐Index (95% CI)^b^
PRS*_APOE_*			
Model 1	PRS*_APOE_*	0.60 (0.55, 0.64)	0.58 (0.55, 0.61)
Model 2	PRS*_APOE,_* age	0.76 (0.72, 0.80)	0.74 (0.72, 0.77)
Model 3	PRS*_APOE,_* age, sex	0.79 (0.75, 0.82)	0.78 (0.76, 0.81)
Model 4	PRS*_APOE,_* age, sex, education	0.80 (0.77, 0.84)	0.80 (0.77, 0.82)
PRS_non_ *_‐APOE_*			
Model 1	PRS_non_ *_‐APOE_*	0.60 (0.55, 0.64)	0.57 (0.54, 0.60)
Model 2	PRS_non_ *_‐APOE_*, age	0.76 (0.72, 0.80)	0.74 (0.72, 0.77)
Model 3	PRS_non_ *_‐APOE_*, age, sex	0.79 (0.75, 0.82)	0.78 (0.76, 0.81)
Model 4	PRS_non_ *_‐APOE_*, age, sex, education	0.81 (0.77, 0.84)	0.80 (0.77, 0.82)

Abbreviations: AUC, area under the receiver operating characteristic curve; CI, confidence interval; MIND‐China, Multimodal Interventions to Delay Dementia and Disability in Rural China; PRS, polygenic risk score; SYS‐AD, Shandong Yanggu Study of Aging and Dementia.

^a^ AUC (95% CI) assessed the ability of different models for discriminating AD from non‐dementia, derived from the analysis of cross‐sectional data in the 2018 MIND‐China study.

^b^ C‐index assessed the ability of different models for predicting AD, derived from the analysis of follow‐up data from 2014 to 2018 in the SYS‐AD cohort study.

**FIGURE 2:**
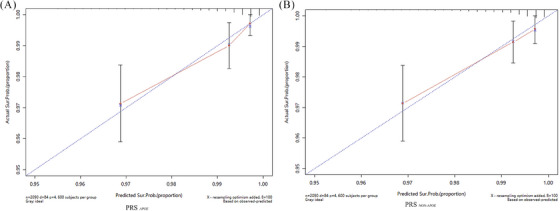
Internal validation of the polygenic risk score (PRS) model for predicting incident Alzheimer's disease (AD) over 3 years, from 2014 to 2015 to 2018. Calibration curves indicate the predicted incident AD over 3 years. (A) PRS*_APOE_*; (B) PRS_non‐_ *_APOE_*. The x‐axis indicates the probability of predicted incident AD, and the y‐axis indicates the actual probability of incident AD. The gray line indicates the agreement between the predicted and actual incident AD. PRS, polygenic risk score

We further performed the bootstrap‐based internal validation of the compound model that included PRS*_APOE_*, age, sex, and education for predicting the incidence of AD. The calibration plots showed that the predicted incidence of AD at 3 years closely corresponded with the actual observed incidence, with the optimism‐corrected C‐statistic being 0.78 and the Brier score being 0.01 (Figure 2A). The predictive ability of the compound model with PRS_non_ *_‐APOE_* was similar to that of PRS*_APOE_*, with the optimism‐corrected C‐statistic being 0.76 and the Brier score being 0.01 (Figure 2B).

### 3.5 Cross‐sectional associations of PRS with plasma Aβ, total tau, and NfL in the 2018 MIND‐China Study (*n* = 1256)

In the subsample of plasma biomarkers from the 2018 MIND‐China Study (*n* = 1256), the general linear regression analysis suggested that the PRS*_APOE_* was significantly associated with a reduced plasma Aβ42/Aβ40 ratio, even in the multivariable‐adjusted model (Table 5). When the PRS*_APOE_* was analyzed as quartiles, the third and fourth quartiles (vs. first quartile) were significantly and marginally associated with reduced plasma Aβ42, respectively. The fourth quartile (Q4 vs. Q1) of the PRS*_APOE_* was significantly associated with a lower plasma Aβ42/Aβ40 ratio and higher plasma NfL (*p* < 0.05). There was no significant association of PRS*_APOE_* with plasma Aβ40 or t‐tau (*p* > 0.05) (Table 5). PRS_non‐_ *_APOE_* was not significantly associated with any of the examined plasma AD biomarkers (*p* > 0.05) (Table S2).

**TABLE 5 alz13924-tbl-0005:** Associations of polygenic risk score (PRS) with Alzheimer's disease plasma biomarkers (*n*  = 1256).

PRS with *APOE* gene (PRS*_APOE_*)	No. of subjects	β‐coefficient (95% confidence interval), plasma biomarkers	
		Mode1 1^a^	Mode1 2^a^
Aβ42 (pg/mL)			
PRS*_APOE_*, continuous	1256	−0.299 (‐0.641, 0.043)	−0.305 (‐0.648, 0.038)
PRS*_APOE_* (quartiles)			
Q1 (<‐1.261)	314	0.000 (reference)	0.000 (reference)
Q2 (‐1.261– ‐0.983)	317	−0.053 (‐0.513, 0.407)	−0.065 (‐0.527, 0.396)
Q3 (‐0.982– ‐0.674)	311	−0.526 (‐0.989, ‐0.064)^*^	−0.552 (‐1.015, ‐0.09)^*^
Q4 (> ‐0.674)	314	−0.400 (‐0.862, 0.062)	−0.414 (‐0.877, 0.049)
*p* for linear trend		0.025	0.021
Aβ40 (pg/mL)			
PRS*_APOE_*, continuous	1256	0.283 (‐5.230, 5.795)	0.276 (‐5.243, 5.795)
PRS*_APOE_* (quartiles)			
Q1 (<‐ 1.261)	314	0.000 (reference)	0.000 (reference)
Q2 (‐1.261– ‐0.983)	317	−1.041 (‐8.467, 6.384)	−1.146 (‐8.583, 6.291)
Q3 (‐0.982– ‐0.674)	311	−4.105 (‐11.566, 3.356)	−4.487 (‐11.939, 2.965)
Q4 (> ‐0.674)	314	−0.131 (‐7.584, 7.322)	−0.156 (‐7.615, 7.304)
*p* for linear trend		0.773	0.750
Aβ42/Aβ40 ratio (×100)			
PRS*_APOE_*, continuous	1256	−0.194 (‐0.383, ‐0.005)^*^	−0.195 (‐0.384, ‐0.006)^*^
PRS*_APOE_* (quartiles)			
Q1 (<‐1.261)	314	0.000 (reference)	0.000 (reference)
Q2 (‐1.261– ‐0.983)	317	−0.031 (‐0.286, 0.223)	−0.038 (‐0.293, 0.217)
Q3 (‐0.982– ‐0.674)	311	−0.112 (‐0.368, 0.144)	−0.112 (‐0.367, 0.143)
Q4 (> ‐0.674)	314	−0.256 (‐0.511, 0.010)	−0.261 (‐0.517, ‐0.005)^*^
*p* for linear trend		0.040	0.037
Total tau (pg/mL)			
PRS*_APOE_*, continuous	1256	−0.009 (‐0.064, 0.046)	−0.012 (‐0.066, 0.043)
PRS*_APOE_* (quartiles)			
Q1 (<‐1.261)	314	0.000 (reference)	0.000 (reference)
Q2 (‐1.261– ‐0.983)	317	−0.015 (‐0.088, 0.059)	−0.019 (‐0.093, 0.055)
Q3 (‐0.982– ‐0.674)	311	0.030 (‐0.044, 0.103)	0.028 (‐0.046, 0.102)
Q4 (> ‐0.674)	314	0.012 (‐0.061, 0.086)	0.009 (‐0.065, 0.083)
*p* for linear trend		0.492	0.535
NfL (pg/mL)			
PRS*_APOE_*, continuous	1255	0.052 (‐0.007, 0.111)	0.057 (‐0.001, 0.116)
PRS*_APOE_* (quartiles)			
Q1 (<‐ 1.261)	314	0.000 (reference)	0.000 (reference)
Q2 (‐1.261– ‐0.983)	316	0.032 (‐0.047, 0.111)	0.031 (‐0.048, 0.109)
Q3 (‐0.982– ‐0.674)	311	0.016 (‐0.064, 0.095)	0.014 (‐0.065, 0.093)
Q4 (> ‐0.674)	314	0.080 (0.000, 0.159)^*^	0.085 (0.005, 0.164)^*^
*p* for linear trend		0.082	0.063

Abbreviations: AD, Alzheimer's disease; Aβ, amyloid β; NfL, neurofilament light chain; PRS, polygenic risk score.

^a^ Model 1 was adjusted for age and sex, and model 2 was adjusted for age, sex, education, smoking, alcohol drinking, hypertension, hyperlipidemia, diabetes, coronary heart disease, and stroke. Plasma total tau and NfL concentrations were log transformed.

^*^ *p *< 0.05.

## 4 DISCUSSION

In this community‐based study of rural Chinese older adults, we explored the cross‐sectional and longitudinal associations of PRS with and without *APOE* gene with AD, evaluated its discriminative and predictive ability for AD, and explored its associations with AD‐related plasma biomarkers. We found that the PRS*_APOE_* was associated both cross‐sectionally and longitudinally with a nearly two‐fold increased risk of AD and that the PRS*_APOE_* in combination with age, sex, and education showed good discriminative and predictive accuracy for AD. In support of these observations, PRS*_APOE_* was associated with reduced plasma Aβ42 and the Aβ42/40 ratio. When *APOE* gene was excluded from the PRS, similar associations with AD, but not with AD‐related plasma biomarkers, were observed. To the best of our knowledge, this is the first community‐based study in a Chinese population to explore the association of PRS with and without *APOE* gene with AD and plasma biomarkers for amyloid and neurodegeneration.[Table alz13924-tbl-0005]

Previously, population‐based studies have shown a 1.3‐ to 2.32‐fold increased risk of AD associated with per one‐unit increase in PRS.^7^, ^26^, ^27^ However, the large majority of these studies have been conducted among European populations. A clinical‐based study in China showed that PRS was associated with a 1.58‐fold increased likelihood of AD.^14^ Our population‐based cohort study suggested that the PRS (per 1‐point increase) was associated with a nearly two‐fold increased risk of developing AD. Differences in the study settings (e.g., clinical vs. the general population setting), characteristics of the study populations (e.g., sex or gender, race, and education), and the SNPs selected for the development of PRS might partially contribute to the discrepancies across studies. Of note, a considerable proportion (~40%) of participants in our study sample (who were born before the early 1950s) had no formal education, especially for women (85.4% women vs. 14.6% men had no formal school education, *p* < 0.001). We previously reported that education was a primary proxy of cognitive reserve and that greater cognitive reserve was associated with a reduced risk of dementia and mild cognitive impairment, even among older adults with no or limited formal education, possibly by compensating for the impact of neurodegeneration.^28^, ^29^ This might partly explain the interaction between sex and PRS*_APOE_* on AD detected in our study, such that women with a high PRS*_APOE_* had a substantially increased likelihood of AD than men with a low PRS*_APOE_*. Findings from our study also contribute to the generalizability of PRS for AD in the context of demographically, ethnically, and geographically diverse populations.^30^ Furthermore, our study showed that PRS with and without *APOE* gene was similarly associated with AD in both cross‐sectional and longitudinal analyses. Previous research has suggested that *APOE* ε4 allele, as the strongest genetic risk factor for late‐onset AD, can explain around 20% of the late‐onset AD risk in Caucasian ancestry^31^, and that *APOE* ε4 alone was stronger in predicting AD risk than the combination of all non‐*APOE* common variants.^7^, ^32^, ^33^, ^34^ However, the *APOE* ε4 allele was not evidently associated with an increased risk of AD in our Chinese cohort. Indeed, evidence has suggested that the *APOE* ε4 allele was differentially associated with AD risk across various ethnic populations.^35^, ^36^ The association of *APOE* ε4 allele with AD was relatively weaker in East Asians than in Caucasians. ^37^ In line with our study, the community‐based Shanghai Aging Study found no evident association between *APOE* ε4 allele and AD risk^38^, ^39^, although the meta‐analysis of case‐control studies did report an association between *APOE* ε4 allele and AD among Chinese populations. The potential selection bias of hospital‐based case‐control studies might partly contribute to the discrepant findings.

Our study showed that the discriminative and predictive ability of PRS alone for AD was poor, with the AUC being 0.60 and the C‐index being 0.58, which was generally in line with reports from previous studies.^7^, ^13^, ^14^ This supports the view that the utility of the PRS alone in the discrimination and risk prediction of AD is limited over demographic factors such as age and education.^10^ The discriminative and predictive ability of PRS alone for AD was similar to that of PRS for other diseases (e.g., stroke or cancer).^40^, ^41^ This is in line with the fact that the late‐life risk of multifactorial diseases such as AD is eventually determined by genetic susceptibility, lifelong environmental factors, and their interactions.^42^ However, the discriminative and predictive ability of PRS for AD was substantially improved when being combined with demographic factors (i.e., age, sex, and education), with the AUC being 0.81 and C‐index being 0.80. The predictive ability of the PRS plus demographics for AD was internally validated; however, our discriminative and predictive models for AD deserve external validation among Chinese populations in future studies.

The associations of PRS with AD‐related plasma biomarkers provide compelling support for the association of the PRS with AD. We found that a higher PRS*_APOE_* was associated with reduced plasma Aβ42 concentration and the Aβ42/Aβ40 ratio, which was consistent with a clinical‐based study from China.^13^ Previous studies have demonstrated that plasma Aβ42 and the Aβ42/Aβ40 ratio measured using the SIMOA technology were correlated with those biomarkers in CSF^7^ and the brain.^43^ Moreover, a low plasma Aβ42/40 ratio was predictive of future accumulation of Aβ in the brain.^44^ These results indicate that PRS*_APOE_* may modulate genetic risk of AD via Aβ pathology. However, we found no association of PRS*_non‐APOE_* with plasma Aβ42 and the Aβ42/Aβ40 ratio. This suggests that the association of PRS*_APOE_* with increased plasma Aβ42 and the reduced Aβ42/Aβ40 ratio might be driven primarily by *APOE* gene, which is consistent with a previous study.^7^ Plasma NfL was a reliable biomarker for neuronal injury and unspecific neurodegeneration, one of the key pathological features of AD. We found that a higher PRS*_APOE_* was associated with higher plasma NfL, which was in line with a clinical‐based study in China.^13^ Our study again showed no association of PRS*_non‐APOE_* with plasma NfL. However, the Gothenburg H70 Birth Cohort Studies in Sweden found that the PRS without *APOE* gene was associated with NfL in CSF only in a selective group of individuals without Aβ42 pathology.^45^ The discrepancies may be due partly to differences in the target populations as well as in SNPs used to develop PRS. Although the association of PRS*_APOE_* with clinical AD was similar to that of the non‐*APOE* PRS, our study did show that PRS*_APOE_* appeared to better reflect the AD‐related neuropathology.

This is the first population‐based study to examine the PRS with and without *APOE* gene in association with AD and related plasma biomarkers in a Chinese population that engaged rural older adults in China, a sociodemographic group that is disproportionately affected by dementia but has been substantially underrepresented in Alzheimer's research^46^, ^47^. However, our study has limitations. First, this was a single‐center study with a relatively short follow‐up period. Furthermore, most of the SNPs were derived from previous GWAS studies that targeted Caucasian populations. As a result, some of the risk loci that are unique to the Chinese population (e.g., SNPs rs3777215, rs6859823, rs234434, and rs2255835) might have been missed.^48^

In summary, the PRS with and without *APOE* gene generated from common variants was both cross‐sectionally and longitudinally associated with an elevated risk of AD among rural older adults in China. The PRS in combination with demographic factors (i.e., age, sex, and education) could be useful for identifying individuals at high risk for AD. The correlation of the PRS*_APOE_* with AD‐related plasma biomarkers suggests that the AD‐related pathologies may underlie the polygenetic predisposition for AD.

## AUTHOR CONTRIBUTIONS

Tingting Hou: study design, data collection, data interpretation, and writing the first draft of the manuscript. Keke Liu: study design, data analysis, and co‐writing of the manuscript. Cuicui Liu, Wenxin Fa, Liang X.Y, Min Zhu, Lin Cong, Yongxiang Wang, Shi Tang, Xiang Wang, Yifei Ren, Qihuan Tan, and Shan Xu: data collection and interpretation. Yifeng Du and Chengxuan Qiu: study design, data interpretation, and study supervision. All authors: critical revisions and approval of the manuscript.

## CONFLICT OF INTEREST STATEMENT

The authors declare no conflict of interest. Author disclosures are available in the supporting information.

## CONSENT STATEMENT

The study was approved by the Institutional Review Boards at all sites and written informed consent was obtained for all participants. All phases of data collection in the MIND‐China study and SYS‐AD study were approved by the Ethics Committee on Human Experimentation at Shandong Provincial Hospital affiliated with Shandong First Medical University in Jinan, Shandong, China. Written informed consents were obtained from all participants, or if the participants were not able to give consent due to severe cognitive impairment, from the informants.

## Supporting information

Supporting Information

Supporting Information
