# Supplementary material for: Association of polygenic risk scores with Alzheimer's disease and plasma biomarkers among Chinese older adults: A community‐based study
Source: Alzheimers Dement. 2024 Aug 22;20(10):6669–81. doi: 10.1002/alz.13924 (PMC11485307; doi:10.1002/alz.13924)
Supplement: Supplementary file 1 — Supporting Information [file ALZ-20-6669-s002.docx]

**Supplementary materials**

**Association of polygenic risk scores with Alzheimer’s disease among Chinese older adults: a community-based study**

Tingting Hou^1,2,3,4#^, Keke Liu^1,2,3,4#^, Wenxin Fa^1^, Cuicui Liu^1,2,3,4^, Min Zhu^1,2,3,4^, Xiaoyan Liang^2^, Yifei Ren^2^, Shan Xu^2^, Xiang Wang^1,2,3^, Shi Tang^1,2,3,4^, Yongxiang Wang^1,2,3,4,5^, Lin Cong ^1,2,3,4^, Qihuan Tan^6^, Yifeng Du^1,2,3,4*^, Chengxuan Qiu^2,5,6*^

**Authors’ affiliations**

^1^Department of Neurology, Shandong Provincial Hospital Affiliated to Shandong First Medical University, Jinan, Shandong, 250021, P.R. China

^2^Department of Neurology, Shandong Provincial Hospital, Shandong University, Jinan, Shandong, 250021, P.R. China

^3^Shandong Provincial Clinical Research Centre for Neurological Diseases, Jinan, Shandong, 250021, P.R. China

^4^Key Laboratory of Endocrine Glucose & Lipids Metabolism and Brain Aging, Ministry of Education; Department of Neurology, Shandong Provincial Hospital Affiliated to Shandong First Medical University, Jinan, Shandong, 250021, China

^5^Aging Research Center and Center for Alzheimer Research, Department of Neurobiology, Care Sciences and Society, Karolinska Institute-Stockholm University, 17165 Solna, Sweden

^6^ Epidemiology and Biostatistics, Department of Public Health, University of Southern Denmark, 5000, Odense, Denmark

^#^Tingting Hou and Keke Liu contributed equally to this work.

**Contents**

**TABLE S1** Characteristics of the single nucleotide polymorphisms included in the selection process

**FIGURE S1** Histogram distribution of PRS***_APOE_*** (A) and PRS_non_*_-APOE_* (B) among participants in the 2018 MIND-China Study (n=4967)

**TABLE S2** Associations of polygenic risk score without *APOE* gene with Alzheimer’s disease plasma biomarkers

**TABLE S1** Characteristics of the single nucleotide polymorphisms included in the selection process

| NeighboringGene | Single nucleotide polymorphisms | Chr | Location | Minor allele | MAF | | SNP origin, year [reference] | Inclusion in selection |
| --- | --- | --- | --- | --- | --- | --- | --- | --- |
|  |  |  |  |  | Controls | AD |  |  |
| *CR1* | rs6656401* | 1 | \| 207692049 \| \| --- \| | A | 2.97 | 1.93 | Lambert et al., 2013^1^ | YES* |
| *CR1* | rs3818361* | 1 | 207784968 | A | 36.74 | 33.15 | Li et al., 2015^2^ | YES* |
| *CR1* | rs4844610 | 1 | 207802552 | A | 2.83 | 1.93 | [Kunkle](https://pubmed.ncbi.nlm.nih.gov/?sort=date&term=Kunkle+BW&cauthor_id=30820047) et al., 2019^3^ | YES |
| *CELF1* | rs10838725* | 11 | 47557871 | C | 27.65 | 30.39 | Lambert et al., 2013^1^ | YES* |
| *MS4A6A* | rs983392 | 11 | 59923508 | G | 5.37 | 5.25 | Lambert et al., 2013^1^ | YES |
| *MS4A6A* | rs610932 | 11 | 59939307 | T | 29.98 | 29.28 | Tan et al., 2013^4^ | YES |
| *PICALM* | rs10792832 | 11 | 85867875 | A | 36.57 | 38.95 | Lambert et al., 2013^1^ | YES |
| *PICALM* | rs3851179 | 11 | 85868640 | T | 36.56 | 38.95 | [Kunkle](https://pubmed.ncbi.nlm.nih.gov/?sort=date&term=Kunkle+BW&cauthor_id=30820047) et al. 2019^3^ | YES |
| *SORL1* | rs11218343* | 11 | 121435587 | C | 28.39 | 30.94 | Lambert et al., 2013^1^ | YES* |
| *FERMT2* | rs4901317 | 14 | 53390305 | C | 4.80 | 4.97 | Lin et al., 2017^5^ | YES |
| *FERMT2* | rs17125924 | 14 | 53391680 | G | 26.32 | 27.90 | [Kunkle](https://pubmed.ncbi.nlm.nih.gov/?sort=date&term=Kunkle+BW&cauthor_id=30820047) et al. 2019^3^ | YES |
| *FERMT2* | rs17125944 | 14 | 53400629 | C | 23.33 | 24.03 | Lambert et al., 2013^1^ | YES |
| *GCH1* | rs72713460 | 14 | 55297043 | T | 13.89 | 14.92 | Zhou et al., 2018^6^ | YES |
| *SLC24A4* | rs10498633* | 14 | 92926952 | T | 9.43 | 11.60 | Lambert et al., 2013^1^ | YES* |
| *SLC24A4* | rs12881735* | 14 | 92932828 | C | 9.45 | 11.60 | [Kunkle](https://pubmed.ncbi.nlm.nih.gov/?sort=date&term=Kunkle+BW&cauthor_id=30820047) et al., 2019^3^ | YES |
| *LINC01413* | rs2591054 | 14 | 57612410 | T | 28.15 | 30.39 | Zhou et al., 2018^6^ | YES |
| *ADAM10* | rs593742 | 15 | 59045774 | A | 23.60 | 24.03 | [Kunkle](https://pubmed.ncbi.nlm.nih.gov/?sort=date&term=Kunkle+BW&cauthor_id=30820047) et al., 2019^3^ | YES |
| *IQCK* | rs7185636 | 16 | 19808163 | C | 20.25 | 21.55 | [Kunkle](https://pubmed.ncbi.nlm.nih.gov/?sort=date&term=Kunkle+BW&cauthor_id=30820047) et al., 2019^3^ | YES |
| *WWOX* | rs62039712 | 16 | 79355857 | A | 0.24 | 0 | [Kunkle](https://pubmed.ncbi.nlm.nih.gov/?sort=date&term=Kunkle+BW&cauthor_id=30820047) et al., 2019^3^ | NO |
| *ACE* | rs138190086* | 17 | 61538148 | A | 1.36 | 2.21 | [Kunkle](https://pubmed.ncbi.nlm.nih.gov/?sort=date&term=Kunkle+BW&cauthor_id=30820047) et al., 2019^3^ | YES* |
| *ABCA7* | rs3764650 | 19 | 1046520 | G | 28.84 | 31.49 | Liu et al., 2014^7^ | YES |
| *ABCA7* | rs4147929 | 19 | 1063443 | A | 31.83 | 34.53 | Lambert et al., 2013^1^ | YES |
| *PVRL2* | rs6859 | 19 | 45382034 | A | 29.99 | 31.77 | Xiao et al., 2015^8^ | YES |
| *TOMM40* | rs2075650* | 19 | 45395619 | G | 8.55 | 11.33 | Ma et al., 2013^9^ | YES* |
| *apoC1* | rs73052335 | 19 | 45420082 | C | 9.42 | 10.22 | Zhou et al., 2018^6^ | YES |
| *CD33* | rs3865444 | 19 | 51727962 | A | 19.52 | 18.23 | Tan et al., 2013^4^ | YES |
| *Bin* | rs6733839* | 2 | 127892810 | T | 44.69 | 47.79 | Lambert et al., 2013^1^ | YES* |
| *Bin* | rs744373 | 2 | 127894615 | G | 35.51 | 36.74 | Li et al., 2015^10^ | YES |
| *INPP5D* | rs10933431* | 2 | 233981912 | G | 34.36 | 31.22 | [Kunkle](https://pubmed.ncbi.nlm.nih.gov/?sort=date&term=Kunkle+BW&cauthor_id=30820047) et al., 2019^3^ | YES* |
| *INPP5D* | rs35349669 | 2 | 234068476 | T | 1.78 | 2.21 | Lambert et al., 2013^1^ | YES |
| *CASS4* | rs6024870 | 20 | 54997568 | A | 0.16 | 0.28 | [Kunkle](https://pubmed.ncbi.nlm.nih.gov/?sort=date&term=Kunkle+BW&cauthor_id=30820047) et al., 2019^3^ | NO |
| *CASS4* | rs911159 | 20 | 55012318 | A | 15.37 | 13.81 | Liu et al., 2014^7^ | YES |
| *CASS4* | rs7274581 | 20 | 55018260 | C | 0.14 | 0.28 | Lambert et al., 2013^1^ | NO |
| *ADAMTSI* | rs2830500* | 21 | 28156856 | A | 4.36 | 2.76 | [Kunkle](https://pubmed.ncbi.nlm.nih.gov/?sort=date&term=Kunkle+BW&cauthor_id=30820047) et al., 2019^3^ | YES* |
| *KCNJ15* | rs928771 | 21 | 39663760 | G | 17.21 | 16.30 | Zhou et al., 2018^6^ | YES |
| *MEF2C-AS1* | rs9293506* | 5 | 88186671 | T | 9.43 | 11.33 | Liu et al., 2014^7^ | YES* |
| *MEF2C-AS1* | rs190982* | 5 | 88223420 | G | 15.92 | 12.71 | Lambert et al., 2013^1^ | YES* |
| *CD2AP* | rs9473117 | 6 | 47431284 | C | 10.77 | 10.65 | [Kunkle](https://pubmed.ncbi.nlm.nih.gov/?sort=date&term=Kunkle+BW&cauthor_id=30820047) et al., 2019^3^ | YES |
| *CD2AP* | rs9349407 | 6 | 47453378 | C | 10.56 | 11.33 | Xiao et al., 2015[8] | YES |
| *CD2AP* | rs10948363 | 6 | 47487762 | G | 10.86 | 11.33 | Lambert et al., 2013^1^ | YES |
| *NME8* | rs2718058 | 7 | 37841534 | G | 20.51 | 21.82 | Lambert et al., 2013^1^ | YES |
| *ZCWPW1* | rs1476679 | 7 | 100004446 | C | 31.85 | 29.83 | Lambert et al., 2013^1^ | YES |
| *EPHA1* | rs10808026 | 7 | 143099133 | A | 14.64 | 15.50 | [Kunkle](https://pubmed.ncbi.nlm.nih.gov/?sort=date&term=Kunkle+BW&cauthor_id=30820047) et al., 2019^3^ | YES* |
| *EPHA1* | rs11767557 | 7 | 143109139 | C | 15.52 | 15.19 | Carrasquillo et al., 2015^11^ | YES |
| *EPHA1* | rs11771145* | 7 | 143110762 | G | 45.25 | 49.45 | Lambert et al. 2013^1^ | YES |
| *PTK2B* | rs28834970 | 8 | 27195121 | C | 23.16 | 23.2 | Lambert et al., 2013^1^ | YES |
| *PTK2B* | rs73223431 | 8 | 27219987 | T | 22.91 | 22.93 | [Kunkle](https://pubmed.ncbi.nlm.nih.gov/?sort=date&term=Kunkle+BW&cauthor_id=30820047) et al., 2019^3^ | YES |
| *CLU* | rs11136000 | 8 | 27464519 | T | 19.48 | 19.06 | Liu et al., 2014^12^ | YES |
| *CLU* | rs9331896 | 8 | 27467686 | C | 18.90 | 19.06 | Lambert et al., 2013^1^ | YES |
| *TREM2* | rs75932628 | 6 | 41129252 | T | 0 | 0 | Guerreiro et al., 2013^13^ | NO |
| *APOE* | rs429358* | 19 | 45411941 | C | 8.25 | 10.77 |  | YES* |
| *APOE* | rs7412 | 19 | 45412079 | T | 7.41 | 6.63 |  | YES |
| *HLA-DRB5-HLA-DRB1* | rs9271192 | 6 | 32578530 |  | Low quality | | Lambert et al., 2013^1^ | NO |

* The single nucleotide polymorphisms that were associated with an elevated odds ratio of Alzheimer’s disease at p<0.30.

**References**

[1] Lambert JC, Ibrahim-Verbaas CA, Harold D, et al. Meta-analysis of 74,046 individuals identifies 11 new susceptibility loci for Alzheimer's disease. Nat Genet. 2013; 45 (12):1452-1458.doi.org/10.1038/ng.2802.

[2] Li Y, Song D, Jiang Y, et al. CR1 rs3818361 Polymorphism Contributes to Alzheimer's Disease Susceptibility in Chinese Population. Mol Neurobiol. 2016; 53 (6):4054-4059.doi.org/10.1007/s12035-015-9343-7.

[3] Kunkle BW, Grenier-Boley B, Sims R, et al. Author Correction: Genetic meta-analysis of diagnosed Alzheimer's disease identifies new risk loci and implicates Abeta, tau, immunity and lipid processing. Nat Genet. 2019; 51 (9):1423-1424.doi.org/10.1038/s41588-019-0495-7.

[4] Tan L, Yu JT, Zhang W, et al. Association of GWAS-linked loci with late-onset Alzheimer's disease in a northern Han Chinese population. Alzheimers Dement. 2013; 9 (5):546-553.doi.org/10.1016/j.jalz.2012.08.007.

[5] Lin E, Tsai SJ, Kuo PH, Liu YL, Yang AC, Kao CF Association and interaction effects of Alzheimer's disease-associated genes and lifestyle on cognitive aging in older adults in a Taiwanese population. Oncotarget. 2017; 8 (15):24077-24087.doi.org/10.18632/oncotarget.15269.

[6] Zhou X, Chen Y, Mok KY, et al. Identification of genetic risk factors in the Chinese population implicates a role of immune system in Alzheimer's disease pathogenesis. Proc Natl Acad Sci U S A. 2018; 115 (8):1697-1706.doi.org/10.1073/pnas.1715554115.

[7] Liu G, Li F, Zhang S, et al. Analyzing large-scale samples confirms the association between the ABCA7 rs3764650 polymorphism and Alzheimer's disease susceptibility. Mol Neurobiol. 2014; 50 (3):757-764.doi.org/10.1007/s12035-014-8670-4.

[8] Xiao Q, Liu ZJ, Tao S, et al. Risk prediction for sporadic Alzheimer's disease using genetic risk score in the Han Chinese population. Oncotarget. 2015; 6 (35):36955-36964.doi.org/10.18632/oncotarget.6271.

[9] Ma XY, Yu JT, Wang W, et al. Association of TOMM40 polymorphisms with late-onset Alzheimer's disease in a Northern Han Chinese population. Neuromolecular Med. 2013; 15 (2):279-287.doi.org/10.1007/s12017-012-8217-7.

[10] Li HL, Yang P, Liu ZJ, et al. Common variants at Bin1 are associated with sporadic Alzheimer's disease in the Han Chinese population. Psychiatr Genet. 2015; 25 (1):21-25.doi.org/10.1097/YPG.0000000000000071.

[11] Carrasquillo MM, Crook JE, Pedraza O, et al. Late-onset Alzheimer's risk variants in memory decline, incident mild cognitive impairment, and Alzheimer's disease. Neurobiol Aging. 2015; 36 (1):60-67.doi.org/10.1016/j.neurobiolaging.2014.07.042.

[12] Liu G, Wang H, Liu J, et al. The CLU gene rs11136000 variant is significantly associated with Alzheimer's disease in Caucasian and Asian populations. Neuromolecular Med. 2014; 16 (1):52-60.doi.org/10.1007/s12017-013-8250-1.

[13] Ruiz A, Dols-Icardo O, Bullido MJ, et al. Assessing the role of the TREM2 p.R47H variant as a risk factor for Alzheimer's disease and frontotemporal dementia. Neurobiol Aging. 2014; 35 (2):444 e441-444.doi.org/10.1016/j.neurobiolaging.2013.08.011.


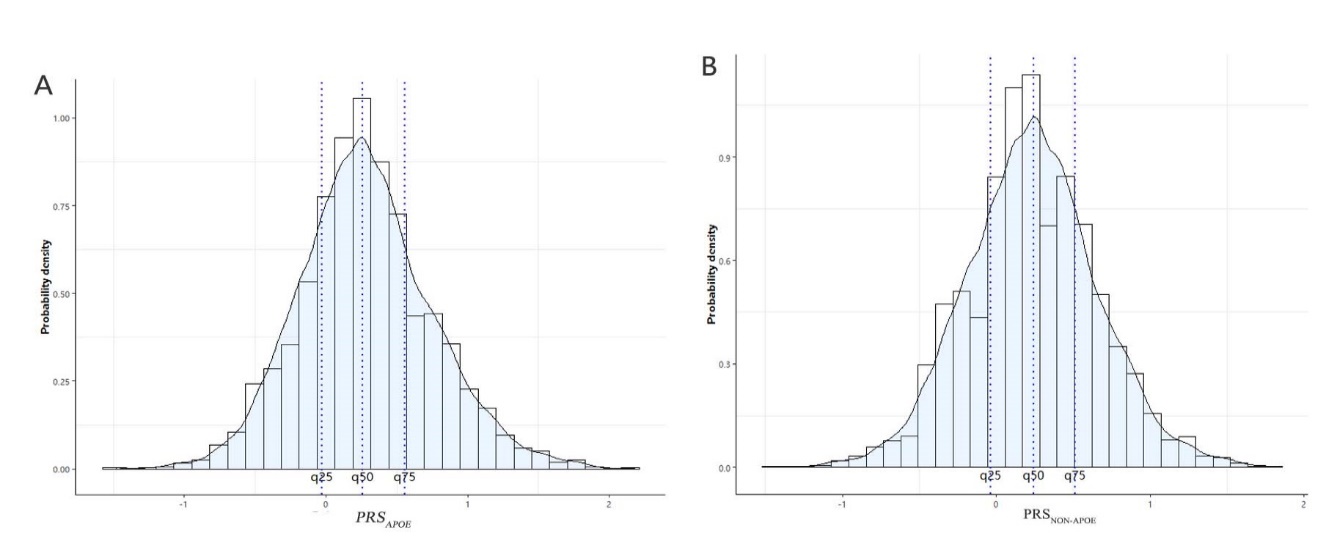


**FIGURE S1** Histogram distribution of PRS*_APOE_* (A) and PRS_non_*_-APOE_* (B) among participants in the 2018 MIND-China Study (n=4967)

The vertical lines show the cut-off values for the quartiles of polygenic risk score: The cut-off for the 25th, 50th, and 75th percentile was -0.030, 0.256, and 0.556, respectively, for PRS*_APOE_*; and -0.036, 0.242, and 0.511, respectively, for PRS_non_*_-APOE_*.

**TABLE S2** Associations of polygenic risk score without *APOE* gene with Alzheimer’s disease plasma biomarkers

| **Polygenic risk score without *APOE* gene (PRS_non_*_-APOE_*)** | **No. of subjects** | **β-coefficient (95% confidence interval), plasma biomarkers** | |
| --- | --- | --- | --- |
|  |  | **Mode1 1^a^** | **Mode1 2^a^** |
| **Aβ42 (pg/ml)** |  |  |  |
| PRS_non_*_-APOE_*, continuous | 1256 | -0.177 (-0.556, 0.202) | -0.185 (-0.565, 0.195) |
| PRS_non_*_-APOE_* (quartiles) |  |  |  |
| Q1 (<-1.269) | 314 | 0.000 (reference) | 0.000 (reference) |
| Q2 (-1.269 - -0.994) | 314 | 0.135 (-0.327, 0.597) | 0.126 (-0.338, 0.589) |
| Q3 (-0.993- -0.726) | 314 | -0.273 (-0.735, 0.189) | -0.297 (-0.759, 0.166) |
| Q4 (>-0.726) | 314 | -0.252 (-0.714, 0.211) | -0.262 (-0.726, 0.201) |
| P for liner trend |  | 0.119 | 0.105 |
| **Aβ40 (pg/ml)** |  |  |  |
| PRS_non_*_-APOE_*, continuous | 1256 | -0.226 (-6.328, 5.877) | -0.403 (-6.511, 5.705) |
| PRS_non_*_-APOE_* (quartiles) |  |  |  |
| Q1 (<-1.269) | 314 | 0.000 (reference) | 0.000 (reference) |
| Q2 (-1.269 - -0.994) | 314 | 1.403 (-6.044, 8.85) | 1.098 (-6.36, 8.555) |
| Q3 (-0.993- -0.726) | 314 | -1.080 (-8.526, 6.365) | -1.546 (-8.99, 5.898) |
| Q4 (>-0.726) | 314 | -1.578 (-9.035, 5.88) | -1.691 (-9.151, 5.770) |
| P for liner trend |  | 0.548 | 0.52 |
| **Aβ42/Aβ40 ratio** (×100) |  |  |  |
| PRS_non_*_-APOE_*, continuous | 1256 | -0.104 (-0.314, 0.106) | -0.099 (-0.309, 0.110) |
| PRS_non_*_-APOE_* (quartiles) |  |  |  |
| Q1 (<-1.269) | 314 | 0.000 (reference) | 0.000 (reference) |
| Q2 (-1.269 - -0.994) | 314 | 0.008 (-0.248, 0.264) | 0.012 (-0.244, 0.268) |
| Q3 (-0.993- -0.726) | 314 | -0.101 (-0.357, 0.154) | -0.096 (-0.351, 0.160) |
| Q4 (>-0.726) | 314 | -0.086 (-0.342, 0.170) | -0.087 (-0.343, 0.169) |
| P for liner trend |  | 0.371 | 0.371 |
| **T-tau (pg/ml)** |  |  |  |
| PRS_non_*_-APOE_*, continuous | 1256 | -0.010 (-0.070, 0.050) | -0.013 (-0.074, 0.047) |
| PRS_non_*_-APOE_* (quartiles) |  |  |  |
| Q1 (<-1.269) | 314 | 0.000 (reference) | 0.000 (reference) |
| Q2 (-1.269 - -0.994) | 314 | -0.0005 (-0.074, 0.073) | -0.007 (-0.081, 0.067) |
| Q3 (-0.993- -0.726) | 314 | 0.046 (-0.028, 0.120) | 0.043 (-0.031, 0.116) |
| Q4 (>-0.726) | 314 | -0.018 (-0.091, 0.056) | -0.021 (-0.095, 0.053) |
| P for liner trend |  | 0.964 | 0.906 |
| **NFL (pg/ml)** |  |  |  |
| PRS_non_*_-APOE_*, continuous | 1255 | 0.055 (-0.010, 0.120) | 0.059 (-0.006, 0.123) |
| PRS_non_*_-APOE_* (quartiles) |  |  |  |
| Q1 (<-1.269) | 314 | 0.000 (reference) | 0.000 (reference) |
| Q2 (-1.269 - -0.994) | 313 | 0.030 (-0.049, 0.110) | 0.028 (-0.051, 0.107) |
| Q3 (-0.993- -0.726) | 314 | 0.052 (-0.027, 0.131) | 0.049 (-0.030, 0.127) |
| Q4 (>-0.726) | 314 | 0.058 (-0.021, 0.138) | 0.063 (-0.016, 0.142) |
| P for liner trend |  | 0.124 | 0.100 |

^a^Model 1 was adjusted for age and sex, and model 2 was adjusted for age, sex, education, smoking, alcohol drinking, hypertension, hyperlipoidemia, diabetes, coronary heart disease, and stroke. ^*^*p*<0.05. Plasma total tau and NFL concentrations were log transformed.

Abbreviations: AD, Alzheimer’s disease; PRS, polygenic risk score; Aβ, amyloid β; NFL, neurofilament light chain.
